# Association of etiological factors across the extreme end and continuous variation in disordered eating in female Swedish twins

**DOI:** 10.1017/S0033291719003672

**Published:** 2021-04

**Authors:** Lisa Dinkler, Mark J. Taylor, Maria Råstam, Nouchine Hadjikhani, Cynthia M. Bulik, Paul Lichtenstein, Christopher Gillberg, Sebastian Lundström

**Affiliations:** 1 Gillberg Neuropsychiatry Centre, Institute of Neuroscience and Physiology, University of Gothenburg, Gothenburg, Sweden; 2 Department of Medical Epidemiology and Biostatistics, Karolinska Institutet, Stockholm, Sweden; 3 Department of Clinical Sciences Lund, Lund University, Lund, Sweden; 4 Athinoula A. Martinos Center for Biomedical Imaging, Massachusetts General Hospital, Harvard Medical School, Charlestown, Massachusetts, USA; 5 Department of Psychiatry, University of North Carolina at Chapel Hill, Chapel Hill, North Carolina, USA; 6 Department of Nutrition, University of North Carolina at Chapel Hill, Chapel Hill, North Carolina, USA; 7 Centre for Ethics, Law and Mental Health (CELAM), Institute of Neuroscience and Physiology, University of Gothenburg, Gothenburg, Sweden

**Keywords:** Adolescence, anorexia nervosa, eating disorders, quantitative genetics, twin study

## Abstract

**Background:**

Accumulating evidence suggests that many psychiatric disorders etiologically represent the extreme end of dimensionally distributed features rather than distinct entities. The extent to which this applies to eating disorders (EDs) is unknown.

**Methods:**

We investigated if there is similar etiology in (a) the continuous distribution of the Eating Disorder Inventory-2 (EDI-2), (b) the extremes of EDI-2 score, and (c) registered ED diagnoses, in 1481 female twin pairs at age 18 years (born 1992–1999). EDI-2 scores were self-reported at age 18. ED diagnoses were identified through the Swedish National Patient Register, parent-reported treatment and/or self-reported purging behavior of a frequency and duration consistent with DSM-IV criteria. We differentiated between anorexia nervosa (AN) and other EDs.

**Results:**

The heritability of the EDI-2 score was 0.65 (95% CI 0.61–0.68). The group heritabilities in DeFries–Fulker extremes analyses were consistent over different percentile-based extreme groups [0.59 (95% CI 0.37–0.81) to 0.65 (95% CI 0.55–0.75)]. Similarly, the heritabilities in liability threshold models were consistent over different levels of severity. In joint categorical-continuous models, the twin-based genetic correlation was 0.52 (95% CI 0.39–0.65) between EDI-2 score and diagnoses of other EDs, and 0.26 (95% CI 0.08–0.42) between EDI-2 score and diagnoses of AN. The non-shared environmental correlations were 0.52 (95% CI 0.32–0.70) and 0.60 (95% CI 0.38–0.79), respectively.

**Conclusions:**

Our findings suggest that some EDs can partly be conceptualized as the extreme manifestation of continuously distributed ED features. AN, however, might be more distinctly genetically demarcated from ED features in the general population than other EDs.

## Introduction

Eating disorders (EDs) comprise several psychiatric conditions, including anorexia nervosa (AN) and bulimia nervosa (BN), characterized by persistent disturbance of eating or eating-related behaviors. EDs are associated with significant disability and mortality (American Psychiatric Association, 2013) and have their onset most often at ages 15 to 20 years (Currin, Schmidt, Treasure, & Jick, 2005; Micali, Hagberg, Petersen, & Treasure, 2013; Stice, Marti, & Rohde, 2013; Zerwas et al., 2015). The prevalence of EDs differs by sex (female predominant) and by type of ED (Hudson, Hiripi, Pope, & Kessler, 2007; Preti et al., 2009; Yao et al., 2016). Diagnostic crossover between different EDs is common (Peat, Mitchell, Hoek, & Wonderlich, 2009), which is likely due to similar symptomatology (e.g. purging and body dissatisfaction) and shared genetic factors (Bulik et al., 2010).

Traditionally, psychiatric disorders have been conceptualized as discrete entities with a clear demarcation between affected and unaffected individuals. However, accumulating evidence suggests that many psychiatric disorders represent the extreme end of dimensionally distributed traits with no clear phenotypic or etiological distinction between affected and unaffected individuals (Martin, Taylor, & Lichtenstein, 2018). This has been demonstrated for a range of disorders, including autism spectrum disorder (Colvert et al., 2015; Lundstrom et al., 2012; Robinson et al., 2016), attention-deficit/hyperactivity disorder (ADHD; Demontis et al., 2019; Greven et al., 2016; Larsson, Anckarsater, Rastam, Chang, & Lichtenstein, 2012), depression (Direk *et al*., 2017; Eley, 1997), anxiety disorders (Taylor et al., 2019), and schizophrenia (Zavos et al., 2014). The dimensional susceptibility is believed to be due to an abundance of common genetic variants, with small cumulative effects (Cross-Disorder Group of the Psychiatric Genomics Consortium, 2013; Plomin, Haworth, & Davis, 2009; Sullivan, Daly, & O'Donovan, 2012).

It has so far only been investigated on a *phenotypic* level, whether EDs are best conceptualized as discrete entities or as the extreme end of variation in continuously distributed cognitions and behaviors characteristic of disordered eating (e.g. body dissatisfaction, dieting, binge-eating, compensatory behaviors; hereafter called *ED features*). Taxometric approaches have yielded conflicting results, supporting both categorical and dimensional conceptualizations of EDs (for a review see Keel, Brown, Holland, & Bodell, 2012). Studies using the (newer) mixture modeling approach, which directly compares the fit of categorical, dimensional, and hybrid models, also provided inconsistent results: while Keel, Crosby, Hildebrandt, Haedt-Matt, and Gravener (2013) suggested a hybrid model structure for BN, Luo, Donnellan, Burt, and Klump (2016) found evidence for a dimensional model for EDs.

An additional approach is to investigate on an *etiological* level whether EDs can be conceptualized as a continuum. Both ED diagnoses and ED features are moderately to highly heritable (Baker et al., 2009; Wade et al., 1999; Yilmaz, Hardaway, & Bulik, 2015). Similar heritability estimates for ED diagnoses and ED features do not, however, necessarily imply that the same genetic variants associated with ED diagnoses are also associated with dimensionally distributed ED features (which would be expected under a dimensional model), since different genetic variants could be mainly responsible for the respective heritability. Furthermore, genome-wide association studies suggest a polygenic architecture of AN (Watson et al., 2019), similar – in principle – to most other psychiatric disorders (Sullivan et al., 2012), and potentially supporting a dimensional hypothesis for EDs.

The aim of the present study was to investigate, in a large-scale population-based twin sample, if EDs can be viewed etiologically as the extreme end of continuous variation in ED features, rather than as distinct entities. First, we investigated whether the etiology in the entire distribution of ED features is similar to the etiology in different extreme groups of ED features. Second, we estimated the genetic correlation between ED features and ED diagnoses. Additionally, we estimated this genetic correlation separately for diagnoses of AN and EDs other than AN, as AN has been proposed to differ from other EDs with respect to prevalence (Keel & Klump, 2003; Keski-Rahkonen & Mustelin, 2016), heritability (Bulik et al., 2010; Dellava, Thornton, Lichtenstein, Pedersen, & Bulik, 2011), as well as comorbidity (Hudson et al., 2007).

## Method

### Participants

Participants were part of the Child and Adolescent Twin Study in Sweden (CATSS), an ongoing longitudinal twin study (Anckarsäter et al., 2011). Parents of twins born in Sweden from 1992 onwards are invited to participate in connection with their twins' 9th birthday (CATSS-9, earlier cohorts included 12-year-olds; answering frequency = 76%). When the twins are aged 18, families are contacted again, irrespective of participation at age 9, and asked to fill out a web-based questionnaire (CATSS-18, answering frequency = 59%). The current study included the birth cohorts 1992–1999. The mean age at CATSS-18 was 18.4 years (s.d. = 0.3, range = 17.9–19.5). Zygosity was either ascertained using a panel of 48 single nucleotide polymorphisms, or an algorithm of five questions regarding twin similarity. CATSS has ethical approval from the Regional Ethical Review Board in Stockholm.

**Fig. 1.**
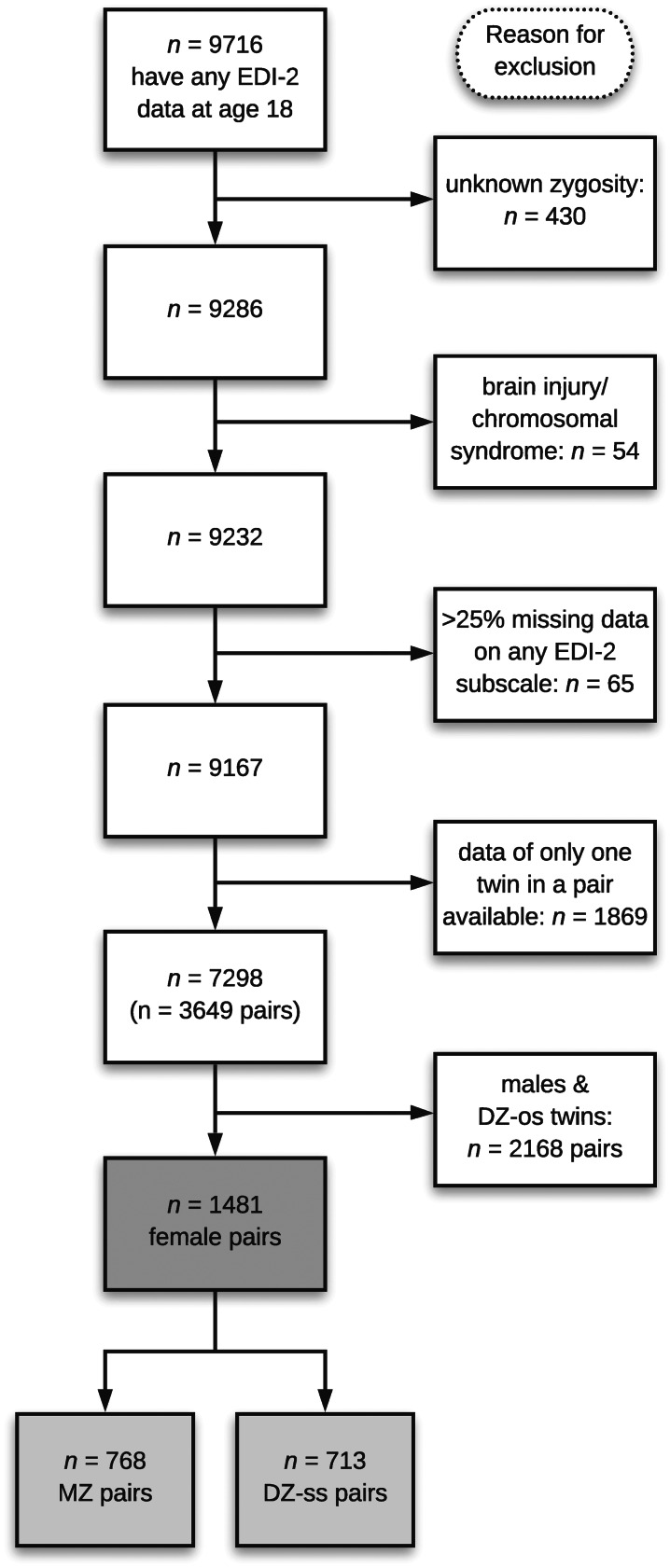
Participant flow chart showing the original and final sample size.

We excluded individuals with congenital or early brain damage syndromes, chromosomal syndromes, unknown zygosity, more than 25% missing data on any of the subscales of the ED feature measurement, and pairs where only one twin in a pair had responded (Fig. 1). We also excluded all males, due to the low number of males identified with a clinical ED (*n* = 20)^1^. The final sample consisted of 1481 female pairs [768 monozygotic (MZ) & dizygotic (DZ) same-sex 713 pairs].

### Measures

#### ED features (continuous measurement)

In CATSS-18, ED features were self-reported on three subscales of the Eating Disorder Inventory-2 (EDI-2; Garner, 1991): *Drive for Thinness* (seven items), *Bulimia* (seven items) and *Body Dissatisfaction* (nine items). Drive for Thinness measures excessive concern with dieting, preoccupation with weight, and fear of weight gain; Bulimia measures the tendency to engage in binge eating and to think about purging; and Body Dissatisfaction measures dissatisfaction with one's overall body shape and the size of specific parts of the body. The response options are ‘never’ (1), ‘rarely’ (2), ‘sometimes’ (3), ‘often’ (4), ‘usually’ (5), and ‘always’ (6). One item on the Body Dissatisfaction scale (‘I like the shape of my buttocks’) was not included in the EDI-2 during the first 6 years of CATSS-18 and therefore only available for 26.2% of the sample; however, the reversed item ‘I think my buttocks are too large’ was included. A total EDI-2 score (hereafter *EDI-2 score*) was computed as the mean of all items on the three subscales when participants had responded to at least 75% of items on each subscale (i.e. at least six out of seven items on Drive for Thinness and Bulimia, and at least seven out of nine items on Body Dissatisfaction). The EDI-2 has been validated in adult Scandinavian females and in non-Swedish adolescents (McCarthy, Simmons, Smith, Tomlinson, & Hill, 2002; Salbach-Andrae et al., 2010). Cronbach's alpha for internal consistency was 0.93 in our sample.

#### ED diagnoses (categorical measurement)

ED diagnoses were identified from three sources. First, we used lifetime diagnoses from the Swedish National Patient Register (NPR), which includes diagnoses from psychiatric inpatient care from 1973 onwards and from specialized outpatient care from 2001 onwards. NPR diagnoses are coded according to ICD-9 and ICD-10. The CATSS data used in this study were linked to the NPR up until 31 December 2016. As a consequence, the end of follow-up in the NPR in our sample was 17 years for the youngest birth cohort (born 1999) and 24 for the oldest birth cohort (born 1992). EDs in the NPR have not yet been subjected to formal reliability/validity testing; however high validity of the NPR has been reported for a range of other psychiatric disorders (Idring et al., 2012; Ludvigsson et al., 2011; Rück et al., 2015; Sellgren, Landen, Lichtenstein, Hultman, & Langstrom, 2011). The following ICD-10 diagnoses of interest were retrieved for all participants: F50.0 (AN), F50.1 (atypical AN), F50.2 (BN), F50.3 (atypical BN), and F50.9 [eating disorder not otherwise specified (EDNOS)]. The Swedish ICD-10 does not provide data on AN subtypes (restricting *v.* binge eating/purging type). It is important to note that the diagnosis of F50.1 (atypical AN) in the ICD system was typically given when the amenorrhea criterion in F50.0 was not present. This is different from the current DSM-5 classification of atypical AN (meets all criteria for AN except low weight). Individuals with DSM-5 atypical AN are not captured by AN/atypical AN in this analysis. We excluded individuals with the following codes: overeating associated with other psychological disturbances (F50.4), vomiting associated with other psychological disturbances (F50.5), other eating disorders (F50.8), feeding disorder of infancy and childhood (F98.2), and Pica of infancy and childhood (F98.3). In total, 93 out of 2962 individuals (3.1%) had an ED diagnosis of interest in the NPR (Table 1).

**Table 1.** Demographic data and prevalence of EDs in the full sample

	MZ		DZ		Total	
	*n*	%	*n*	%	*n*	%
	1536	100	1426	100	2962	100
Birth year						
1992–1993	350	22.8	272	19.1	622	21.0
1994–1995	464	30.2	358	25.1	822	27.8
1996–1997	362	23.6	378	26.5	740	25.0
1998–1999	360	23.4	418	29.3	778	26.3
Birth country of parents						
Both born in Nordic countries^a^	1252	81.5	1160	81.3	2412	81.4
One born in Nordic countries^b^	140	9.1	138	9.7	278	9.4
Both born in other countries	86	5.6	46	3.2	132	4.5
*ED diagnoses*						
1. NPR						
AN	24	1.6	25	1.8	49	1.7
Atypical AN	2	0.1	0	0.0	2	0.07
BN	4	0.3	3	0.2	7	0.2
Atypical BN	1	0.07	0	0.0	1	0.03
EDNOS	44	2.9	23	1.6	67	2.3
Any ED	55	3.6	38	2.7	93	3.1
2. Parent-reported ED treatment^c^	29	1.9	19	1.3	48	1.6
Treatment for AN	27	1.8	19	1.3	46	1.6
Treatment for BN	5	0.3	3	0.2	8	0.3
3. Self-reported regular purging	50	3.3	46	3.2	96	3.2
Total identified cases^d^						
ED	98	6.4	71	5.0	169	5.7
AN	39	2.5	31	2.2	70	2.4
OED	59	3.8	40	2.8	99	3.3

AN, anorexia nervosa; BN, bulimia nervosa; EDNOS, eating disorder not otherwise specified; CATSS, Child and Adolescent Twin Study in Sweden; OED, other eating disorder; MZ, monozygotic twins; DZ, dizygotic twins.

a The Nordic countries include Sweden, Denmark, Finland, Iceland, and Norway and their associated territories (Greenland, the Faroe Islands and the Åland Islands). In 4.7% of cases, birth country information was missing for both parents.

b This includes parents where birth country information was available for one parent only and this parent was born in Sweden.

c Parent-reports of potential ED treatment were available for 72.5% of the total sample (*n* = 2147 for AN, *n* = 2149 for BN).

d Cases were identified through 1. a diagnosis in the NPR, 2. parent-reported ED treatment, and 3. self-reported regular purging.

Second, we used parent-reports of treatment for ED to identify individuals with ED. In CATSS-18, the parents of twins were asked: ‘*Has the twin been treated for AN?*’ and ‘*Has the twin been treated for BN?*’. If parents responded ‘*Yes, earlier*’ or ‘*Yes, now*’ to one or both of these questions, the twin was identified with having/having had an ED. Parent-reported treatment data were available for 72.5% of the sample. Forty-eight individuals were identified with ED; of those, 46 were treated for AN and eight were treated for BN (six individuals were treated for both AN and BN; Table 1). Twenty-six of the 46 individuals with parent-reported treatment for AN also had a diagnosis of AN in the NPR (56.5%). Only one of the eight individuals with parent-reported treatment for BN also had a diagnosis of BN in the NPR (12.5%).

Third, we used self-reports of purging behavior to identify individuals with ED (available for 99.9% of the sample). In CATSS-18, the twins were asked: ‘*Did you ever use vomiting, laxatives, diuretics, or enemas to lose weight or to control your weight?*’. If they responded ‘*Yes, repeatedly over at least three months*’ or ‘*Yes, repeatedly over the last three months*’ they were identified as having/having had an ED (*n* = 96, 3.2%; Table 1). This procedure was deemed to be valid, since the answer options used for identification correspond closely to the DSM-IV criteria for AN binge-eating/purging type (‘regularly engaged in binge-eating or purging behavior’) and BN (‘binge eating and inappropriate compensatory behaviors both occur, on average, at least twice a week for 3 months’), and individuals with repeated purging over at least 3 months would likely receive an ED diagnosis if assessed in a clinical context. When matched to the NPR, 33% of those with regular purging behavior also had an ED diagnosis in the NPR.

The total number of individuals identified with any ED by registered diagnoses, parent-reported treatment and self-reported purging was *n* = 169 (5.7%, Table 1). More than half (55.0%) of all individuals with ED were identified through the NPR. One third (33.7%) of individuals with ED were identified through more than one method. In a final step, we differentiated EDs into AN (including NPR diagnoses of AN and atypical AN, and parent-reported treatment for AN, *n* = 70) and other EDs (OEDs, including NPR diagnoses of BN, atypical BN, and EDNOS, as well as parent-reported treatment for BN and self-reported purging behavior, *n* = 99; Table 1). Individuals diagnosed with both AN and OEDs were included in the AN group. In order to investigate attrition, we compared NPR diagnoses of EDs between our sample and those females who only participated at baseline (age 9), but not at follow-up (age 18, *n* = 2251, ca. 43% of the baseline sample). The prevalence of EDs was very similar in responders and non-responders (3.1% and 3.3%, χ^2^ = 0.15, *p* = 0.70), suggesting that attrition did not bias the representativeness of our sample.

### Statistical analysis

Data analysis was performed using Stata 15.0 (StataCorp, 2017) and OpenMx version 2.12.2 (Neale et al., 2016) in R 3.5.3.

#### The twin design

The twin design is based on comparing the phenotypic resemblance of MZ twins with that of DZ twins. While MZ twins share all of their segregating alleles, DZ twins share on average 50%. The twin model decomposes variance in a trait into additive genetic effects (A), indicated by higher correlations in MZ pairs than in DZ pairs; non-additive genetic effects (D), indicated when MZ correlations are more than twice as high as DZ correlations; shared environment (C), indicated by DZ correlations more than half as high as the MZ correlations; and non-shared environment (E), evidenced by differences within MZ pairs (and including measurement error). The principles of the twin design are described extensively elsewhere (Plomin, DeFries, Knopik, & Neiderhiser, 2013; Posthuma et al., 2003).

#### Full sample heritability of EDI-2 score

We initially fitted a fully saturated model to the observed data, including means, variances, and covariances, to act as a baseline comparison model when comparing model fits. This model was then used to test the assumptions of the twin model. Assumption testing revealed that means and variances could be equated across twin order and zygosity (online Supplementary Table S1). Twin correlations were estimated by zygosity from a constrained saturated model, in which the means and variances were equated within twin pairs and across zygosity. We then fitted a univariate model to estimate the degree of genetic and environmental influences on the EDI-2 score in the entire sample. The DZ twin correlation was slightly larger than half of the MZ twin correlation, therefore we fitted an ACE model. We tested the significance of individual parameters by constraining them to be equal zero. The best-fitting models were chosen based on the likelihood ratio test (if model fit did not deteriorate significantly, the reduced model was favored). In addition, we used the Bayesian information criterion (BIC; lower BIC values indicate better model fit), since the likelihood ratio test can become oversensitive to small deteriorations in model fit when samples are large and may not select the most appropriate model when data are skewed (Derks, Dolan, & Boomsma, 2004; Markon & Krueger, 2004).

#### Extremes analyses of EDI-2 score

We used two different analytic techniques to investigate, whether the etiology of the EDI-2 score was consistent across the entire sample and among those showing extreme EDI-2 scores: DeFries–Fulker extremes analysis and liability threshold modeling. Extreme scorers (probands) were defined using percentile-based cut-offs on the EDI-2, that is, scoring within the 1st, 3rd, 5th, and 10th percentile of the EDI-2 (see the number of probands per percentile in Table 2), in order to maximize power while also capturing individuals with high symptom severity and significant impairment (Robinson et al., 2011).

**Table 2.** Extremes analyses: number of probands, transformed co-twin means and proband-wise concordance rates by EDI-2 percentile

		Transformed co-twin means in DeFries–Fulker extremes analyses^a^		Proband-wise concordance rates in liability threshold models	
EDI-2 percentile	No. probands	MZ	DZ	MZ	DZ
10%	290	0.62	0.32	0.38	0.25
5%	157	0.65	0.32	0.35	0.17
3%	92	0.62	0.31	0.32	0.04
1%	29	0.59	0.30	–	–

MZ, monozygotic twins; DZ, dizygotic twins.

The number of probands applies to both extremes analyses: DeFries–Fulker extremes analyses and liability threshold models.

a In DeFries–Fulker extremes analysis, the scores are transformed so that the population mean is zero, and the proband mean is 1. Transformed co-twin means are interpreted in a similar manner to twin correlations.

DeFries–Fulker extremes analysis assesses consistency in the etiology of a given trait across different severity levels (here the 1st, 3rd, 5th, and 10th percentile of the EDI-2 score) by modeling an individual's expected score as a function of their co-twin's proband status (DeFries & Fulker, 1985; Purcell & Sham, 2003). The twins' EDI-2 scores were transformed so that the population mean was zero and the proband mean was 1. The transformed co-twin means are interpreted in a similar manner to twin correlations. DeFries–Fulker analysis seeks to estimate group heritability (*h*^2^_g_), which estimates the degree to which the genetic influences on extreme scores also influence continuous variation in the same trait. In the classical procedure, a regression equation is fitted to estimate *h*^2^_g_; the equation predicts co-twin scores from the continuous scores of probands. Zygosity is also included as a predictor in the equation; the regression of zygosity on co-twin scores is an estimate of *h*^2^_g_. Here we used a model fitting implementation of the procedure (Purcell & Sham, 2003).

In a second step, liability threshold models were used to estimate the etiology of categorically defined extreme scores on the EDI-2. The liability threshold model is based on dichotomous data, but assumes an underlying continuous distribution of liability to the categorical construct. Probandwise concordance rates were calculated as 2 × (number of concordant pairs)/[2 × (number of concordant pairs) + number of discordant pairs], indicating the probability that a co-twin of a proband is also a proband (Table 2). Using liability threshold models, we estimated the proportion of variation in the liability to extreme ED features that was genetic and environmental at the 1st, 3rd, 5th, and 10th percentile of the EDI-2 score.

#### Joint categorical-continuous models of EDI-2 score and ED diagnoses

We used joint categorical-continuous models to estimate the degree to which genetic influences on the EDI-2 score overlapped with genetic influences on AN and OED diagnoses. The joint categorical-continuous model is a hybridization of a liability threshold model (here for AN/OED diagnoses) with a model for a continuous variable (here for EDI-2 score). Initially, the correlations between EDI-2 score in one twin and ED diagnosis in their co-twin were estimated. If these cross-twin cross-trait correlations are greater in MZ than in DZ twins, genetic influence on the covariation of the EDI-2 score with AN/OED diagnoses is suggested. We then fitted a joint categorical-continuous model; this model allowed us to estimate the genetic, shared environmental, and non-shared environmental correlations between EDI-2 score and AN/OED diagnoses. The genetic correlation (*r*_g_) estimates the degree to which genetic influences on one phenotype are shared with those on another phenotype. A genetic correlation of 1.0 (0.0) indicates that all (none) of the additive genetic influence on two phenotypes is shared between them. The genetic correlation, and the heritability of each trait, can be used to calculate *bivariate heritability*; this refers to the degree to which the genetic factors that overlap across two traits explain the correlation between them. Like the liability threshold model, the categorical component of this model assumes a normal distribution of continuous liability underlying ED diagnosis. In line with the univariate models, we fitted an ACE and its nested models.

## Results

### Descriptive statistics

The mean EDI-2 score was 2.66 (s.d. = 0.90, range = 1–6). The distribution of EDI-2 scores was only slightly skewed (skew = 0.57), nevertheless we transformed the EDI-2 score with the natural logarithm in order to improve the accuracy of the results, as the interpretability of raw scores was not important for the purpose of this study. The prevalence of EDs by and across zygosity is presented in Table 1.

### Full sample heritability of EDI-2 score

**Fig. 2.**
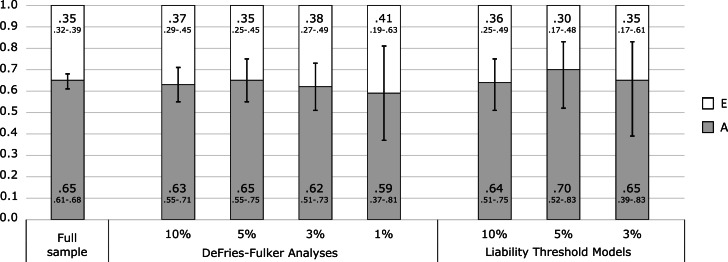
Variance component estimates in the full sample, the DeFries–Fulker extremes analyses and the liability threshold models. Extreme groups (probands) were defined using percentile-based cut-offs on the Eating Disorder Inventory-2 (1st, 3rd, 5th, and 10th percentile). Due to low power, the 1st percentile was not used in the liability threshold models. The numbers in larger font size within bars indicate the estimates; the numbers in smaller font size below each estimate indicate the 95% confidence interval for this estimate. Error bars visualize the 95% confidence intervals for the additive genetic contribution. A, additive genetic contribution; E, non-shared environmental contribution.

The twin correlation in the full sample was higher for MZ than for DZ pairs, suggesting genetic influences on the EDI-2 score (Table 3). According to the likelihood ratio test, the ACE model did not fit significantly worse than the saturated model (Table 4). The best-fitting model was an AE model. The heritability of the EDI-2 score was 0.65 (95% CI 0.61–0.68; Fig. 2).

**Table 3.** Twin correlations in the full sample, the liability threshold models, and the joint categorical-continuous models

	MZ	DZ
Full sample		
	0.65 (0.61–0.68)	0.33 (0.26–0.39)
Liability threshold models (tetrachoric correlations)		
10%	0.64 (0.49–0.76)	0.33 (0.16–0.49)
5%	0.71 (0.52–0.84)	0.30 (0.08–0.50)
3%	0.72 (0.47–0.87)	0.00 (−0.38 to 0.35)
Joint categorical-continuous models		
Cross-twin within-trait		
EDI-2	0.65 (0.62–0.69)	0.33 (0.27–0.39)
ED	0.68 (0.53–0.80)	0.00 (−0.29 to 0.30)
AN	0.71 (0.48–0.86)	−1.00 (NA −0.36)^a^
OED	0.70 (0.51–0.83)	0.28 (−0.07 to 0.58)
Cross-twin cross-trait		
EDI-2 – ED	0.34 (0.23–0.45)	0.08 (−0.07 to 0.24)
EDI-2 – AN	0.26 (0.10–0.41)	0.01 (−0.19 to 0.22)
EDI-2 – OED	0.34 (0.20–0.46)	0.13 (−0.07 to 0.32)

EDI-2, Eating Disorder Inventory-2; ED, any eating disorder; AN, anorexia nervosa; OED, other eating disorder; MZ, monozygotic twins; DZ, dizygotic twins.

a The DZ twin correlation in AN was estimated at −1.00 because there were no DZ pairs concordant for AN. The lower bound of the confidence interval could not be estimated because it was restricted to be −1.00 at minimum.

**Table 4.** Model fit statistics of (a) the ACE and nested models for EDI-2 score in the full sample, (b) the DeFries–Fulker extremes analyses for EDI-2 score by threshold (percentile of the EDI-2 score), (c) the liability threshold models for EDI-2 score by threshold (percentile of the EDI-2 score), and (d) the joint categorical-continuous models for EDI-2 score with diagnoses of any ED, AN, and OED

Model	−2LL	Parameters	df	Comparison model	Δχ^2^	Δdf	*p*	AIC	BIC
(a) Full sample									
Fully saturated	7826.84	10	2952	–	–	–	–	1922.84	−13 724.2
ACE	7835.36	4	2958	Fully saturated	8.53	6	0.20	1919.36	−13 759.4
***AE***	***7835.37***	***3***	***2959***	***ACE***	***0.00***	***1***	***0.95***	***1917.37***	***−13 766.7***
CE	7921.36	3	2959	ACE	86.00	1	<0.001	2003.36	−13 680.7
E	8320.04	2	2960	ACE	484.68	2	<0.001	2400.04	−13 289.4
(b) DeFries–Fulker extremes analyses									
10%									
ACE	4939.70	4	2958	–	–	–	–	−976.30	−16 655.10
***AE***	***4939.76***	***3***	***2959***	***ACE***	***0.05***	***1***	***0.82***	***−978.24***	***−16 662.34***
CE	4963.84	3	2959	ACE	24.13	1	<0.001	−954.16	−16 638.26
5%									
ACE	4311.46	4	2958	–	–	–	–	−1604.54	−17 283.34
***AE***	***4311.46***	***3***	***2959***	***ACE***	***0.00***	***1***	***1.00***	***−1606.54***	***−17 290.64***
CE	4330.51	3	2959	ACE	19.05	1	<0.001	−1587.49	−17 271.59
3%									
ACE	3919.11	4	2958	–	–	–	–	−1996.89	−17 675.69
***AE***	***3919.11***	***3***	***2959***	***ACE***	***0.00***	***1***	***1.00***	***−1998.89***	***−17 682.99***
CE	3941.36	3	2959	ACE	22.25	1	<0.001	−1976.64	−17 660.74
1%									
ACE	3364.71	4	2958	–	–	–	–	−2551.29	−18 230.09
***AE***	***3364.71***	***3***	***2959***	***ACE***	***0.00***	***1***	***1.00***	***−2553.29***	***−18 237.39***
CE	3372.38	3	2959	ACE	7.67	1	0.01	−2545.62	−18 229.72
(c) Liability threshold models									
10%									
Fully saturated	1826.47	6	2956	–	–	–	–	−4085.53	−19 753.73
ACE	1831.57	4	2960	Fully saturated	5.10	4	0.28	−4088.43	−19 777.83
***AE***	***1831.58***	***3***	***2961***	***ACE***	***0.01***	***1***	***0.90***	***−4090.42***	***−19 785.12***
CE	1839.59	3	2961	ACE	8.03	1	<0.001	−4082.41	−19 777.11
E	1898.40	2	2962	ACE	66.83	2	<0.001	−4025.60	−19 725.60
5%									
Fully saturated	1175.23	6	2956	–	–	–	–	−4736.77	−20 404.97
ACE	1184.95	4	2960	Fully saturated	9.72	4	0.05	−4735.05	−20 424.45
***AE***	***1184.95***	***3***	***2961***	***ACE***	***0.00***	***1***	***1.00***	***−4737.05***	***−20 431.75***
CE	1193.30	3	2961	ACE	8.35	1	<0.001	−4728.70	−20 423.40
E	1227.86	2	2962	ACE	42.92	2	<0.001	−4696.14	−20 396.14
3%									
Fully saturated	791.06	6	2956	–	–	–	–	−5120.94	−20 789.14
ACE	799.75	4	2960	Fully saturated	8.69	4	0.07	−5120.25	−20 809.65
***AE***	***799.75***	***3***	***2961***	***ACE***	***0.00***	***1***	***1.00***	***−5122.25***	***−20 816.95***
CE	807.35	3	2961	ACE	7.60	1	0.01	−5114.65	−20 809.35
E	819.93	2	2962	ACE	20.18	2	<0.001	−5104.07	−20 804.07
(d) Joint categorical-continuous models									
ED – EDI-2									
Fully saturated	8857.19	24	5897	–	–	–	–	−2936.81	−34 193.7
ACE	8879.87	11	5911	Fully saturated	22.67	14	0.07	−2942.13	−34 273.2
***AE***	***8879.92***	***8***	***5914***	***ACE***	***0.06***	***3***	***1.00***	***−2948.08***	***−34 295.1***
CE	8974.79	8	5914	ACE	94.92	3	<0.001	−2853.21	−34 200.2
E	9408.05	5	5917	ACE	528.18	6	<0.001	−2425.95	−33 788.9
AN – EDI-2									
Fully saturated	8384.85	24	5900	–	–	–	–	−3415.15	−34 687.9
ACE	8406.06	11	5914	Fully saturated	21.21	14	0.10	−3421.94	−34 768.9
***AE***	***8407.42***	***8***	***5917***	***ACE***	***1.36***	***3***	***0.71***	***−3426.58***	***−34 789.5***
CE	8493.16	8	5917	ACE	87.09	3	<0.001	−3340.84	−34 703.7
E	8922.69	5	5920	ACE	516.63	6	<0.001	−2917.31	−34 296.1
OED – EDI-2									
Fully saturated	8509.94	24	5900	–	–	–	–	−3290.06	−34 562.9
ACE	8531.85	11	5914	Fully saturated	21.91	14	0.08	−3296.15	−34 643.2
***AE***	***8531.95***	***8***	***5917***	***ACE***	***0.1***	***3***	***0.99***	***−3302.05***	***−34 665***
CE	8621.22	8	5917	ACE	89.37	3	<0.001	−3212.78	−34 575.7
E	9045.03	5	5920	ACE	513.18	6	<0.001	−2794.97	−34 173.8

−2LL, −2LogLikelihood; df, degrees of freedom; Δχ^2^, difference in −2LL between two models, distributed χ^2^; Δdf, difference in degrees of freedom between two models; *p*, *p*-value from likelihood-ratio tests; AIC, Akaike Information Criterion; BIC, Bayesian information criterion.

*Note*. The best-fitting model is indicated in bold italics in each case.

### Extremes analyses of EDI-2 score

AE models showed the best fit in all DeFries–Fulker extremes analyses (Table 4). The number of probands per percentile and the transformed co-twin means are shown in Table 2. The group heritability estimates in the DeFries–Fulker extremes analysis (0.59–0.65) were significant, consistent over different levels of severity (1st, 3rd, 5th, and 10th percentile of the EDI-2 score), and similar to the heritability estimates in the full sample, therefore indicating genetic continuity between the continuous distribution and the extremes (Fig. 2).

For the liability threshold models, ACE models did not fit significantly worse than saturated models according to the likelihood ratio tests, and they also had lower BIC values. AE models showed the best fit (Table 4). The heritability estimates were consistent over different levels of severity (0.64–0.70; Fig. 2), indicating consistent etiology for different levels of ED feature severity. We did not conduct the liability threshold analysis for the 1st percentile due to low power.

### Joint categorical-continuous models of EDI-2 score and ED diagnoses

**Fig. 3.**
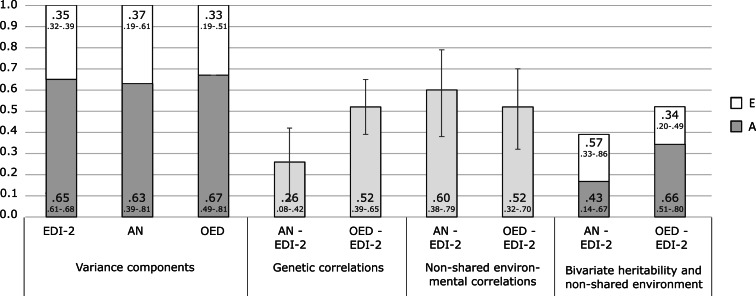
Variance components, correlations, and bivariate estimates from the joint categorical-continuous models. The numbers in larger font size within bars indicate the estimates; the numbers in smaller font size below each estimate indicate the 95% confidence interval for this estimate. Error bars visualize the 95% confidence intervals for the genetic and the non-shared environmental correlations. A, additive genetic contribution; E, non-shared environmental contribution; EDI-2, Eating Disorder Inventory-2 score; AN, anorexia nervosa; OED, other eating disorder.

The cross-twin cross-trait correlations between EDI-2 score and AN/OED diagnoses were more than twice as large in MZ twins compared to DZ twins (Table 3, see the number of concordant and discordant twin pairs in online Supplementary Table S2). This suggests not only additive genetic influence (A), but possibly also non-additive genetic influence (D) on the association between EDI-2 score and AN/OEDs. However, in the univariate model of the EDI-2 score we did not find the influence of D and therefore did not consider it plausible that there would be influence of D on the covariance of EDI-2 score and AN/OEDs. ACE models did not fit significantly worse than saturated models according to the likelihood ratio tests and also had lower BIC values. AE models showed the best fits (Table 4). Heritability was similar for EDI-2 score (*h*^2^ = 0.65), AN (*h*^2^ = 0.63), and OEDs (*h*^2^ = 0.67). The phenotypic correlation was lower for EDI-2 score and AN (*r*_PH_ = 0.39) than for EDI-2 score and OEDs (*r*_PH_ = 0.52) (Fig. 3). Similarly, the genetic correlation was lower for EDI-2 score and AN (*r*_A_ = 0.26) than for EDI-2 score and OEDs (*r*_A_ = 0.52) (online Supplementary Table S3; Fig. 3). Genetic factors accounted for 43% of the correlation between EDI-2 score and AN, and for 66% of the correlation between EDI-2 score and OEDs.

## Discussion

We tested whether EDs can be viewed etiologically as the extreme end of a continuous distribution of ED features rather than as distinct disorders. The etiology of ED features, as measured with the EDI-2, appeared to be consistent across differing severity levels. To a moderate degree, the genetic influences on OED diagnoses also influenced continuous ED features. However, this did not hold true for AN, where the genetic influences on the diagnosis influenced continuous ED features to a lesser extent.

The genetic correlation between ED features and OED diagnoses was moderate. This result implies that OEDs can be conceptualized as the extreme end of dimensionally distributed ED features, rather than as discrete entities. Our findings extend previous evidence from phenotypic studies that EDs are on a continuum (Holm-Denoma, Richey, & Joiner, 2010; Luo et al., 2016; Olatunji et al., 2012; Tylka & Subich, 2003) and they add to the growing body of literature confirming psychiatric disorders as etiologically congruent with dimensional measures of psychopathology (Martin et al., 2018).

For AN, however, the evidence was less strong, as the genetic correlation with ED features was lower than for OEDs. This suggests that the conceptualization of psychiatric disorders as the extreme manifestation of etiologically continuous features might not apply to AN. This result is in line with previous research showing that AN differs from OEDs in a variety of important ways. These include (a) a higher stability of prevalence of AN over time and cultures compared to OEDs (Keel & Klump, 2003; Keski-Rahkonen & Mustelin, 2016), (b) potential differences in risk factors (e.g. childhood adversity; Larsen et al., 2017), (c) different coexisting disorders (Hudson et al., 2007), and (d) the fact that heritability estimates of AN decrease as definitions get broadened (Bulik et al., 2010; Dellava et al., 2011), while this has not been found for BN (Bulik et al., 2010). Furthermore, recent genome-wide association studies suggest that AN may have both psychiatric and metabolic genetic components (e.g. high-density lipoprotein cholesterol, fasting insulin, fasting glucose; Watson et al., 2019). Dimensional measures of ED features may capture the psychiatric components of AN, while they do not cover metabolic factors, which may have lowered the genetic correlation. An important direction for future research will be to evaluate how much of the variance in AN can be attributed to metabolic factors. An alternative explanation for the discontinuity is that the measured dimensions are not the optimal ones to capture underlying dimensional features related to AN; however, it is not entirely clear what more relevant dimensions could be.

The non-shared environmental correlations between ED features and ED diagnoses were above 0.5. This is in contrast to other dimension-disorder relationships such as in autism spectrum disorder and ADHD, where the non-shared environmental correlations are lower (Taylor et al., 2019). In autism spectrum disorder and ADHD, factors such as maternal valproate use during pregnancy and paternal age (D'Onofrio et al., 2014; Modabbernia, Velthorst, & Reichenberg, 2017) might have a stronger relationship with the diagnoses than with the dimensions. In EDs, on the other hand, the higher non-shared environmental correlations between dimensions and diagnoses might reflect psychosocial factors such as pressure for thinness/weight-related teasing, peer dieting behavior, and media exposure to appearance ideals (Culbert, Racine, & Klump, 2015; Mazzeo & Bulik, 2009), which influence both dimensions and diagnoses.

### Implications

Our findings have important implications for future genomic studies of EDs and for how to conceptualize different ED presentations. First, our results suggest that the knowledge derived from studies of ED features may well generalize to diagnosable OEDs, but not necessarily to AN. Second, our data suggest that using ED features in genome-wide association studies may be a viable approach in order to increase sample size and improve statistical power for identifying common genetic variants of OEDs, as has been done for ADHD traits and depressive symptoms in the general population (Demontis et al., 2019; Direk et al., 2017; Middeldorp et al., 2016; Stergiakouli et al., 2015). However, our results indicate that genomic studies of AN should rely on categorical diagnoses rather than dimensional measurements of ED features.

### Strengths and limitations

Our study has several strengths. We had a large sample size and used a validated measure to assess ED features. Uniquely, the linkage of the data with the NPR enabled us to estimate genetic correlations between diagnosed EDs and ED features, including differentiating between AN and OEDs. Additionally, we used parent- and self-reports to compensate for a likely underestimation of ED diagnoses in the NPR. Our findings also have to be considered in the light of several limitations. First, our results apply to females only, as we were underpowered to conduct the analyses for males. Previous research found evidence for quantitative and qualitative sex differences in genetic and environmental influences on ED features, that is, the magnitude of genetic and environmental effects as well as the type of genetic factors involved differs between males and females (Baker et al., 2009). Future studies therefore need to investigate a possible etiological continuum of EDs in males. Second, we were not able to differentiate BN from OEDs, due to the young age of the sample and the later onset of BN compared to AN (Micali et al., 2013; Zerwas et al., 2015). Therefore, studies with longer follow-up time are needed to investigate whether BN can be considered a continuously distributed phenomenon. Third, we were unable to distinguish between AN of the restricting *v.* binge-eating/purging subtype, which precluded our ability to identify factors that would render individuals with AN binge-eating/purging subtype more similar to individuals with BN than those with AN restricting subtype. Related, individuals with self-reported purging and without any NPR diagnosis were collapsed into the OED group; however, a group of these individuals could indeed have had AN of the binge-eating/purging subtype. The effect of this possible misclassification, given the obtained results, would have been an underestimation of the genetic correlation between features and diagnoses of OEDs. Finally, although the overall sample was large, the absolute number of registered ED diagnoses, especially BN, were low. However, we tried to compensate for this by using different methodological approaches as well as including parent- and self-reports in order identify additional cases.

## Conclusion

Our data suggest that a moderate proportion of genetic risks associated with OEDs are also associated with continuous variation in ED features, implying that OEDs can be considered the extreme manifestation of etiologically continuous ED features. Molecular genetic studies of OEDs could benefit from complementing the study of categorically defined OEDs with dimensional ED features, in order to increase statistical power to detect genetic variants. However, the evidence was less strong for AN, suggesting that AN might be more genetically distinct from ED features in the general population compared to OEDs.

## Note

1 We initially investigated sex differences in some of the analyses. Since we had low power for males, we excluded all analyses of males from the paper in order to improve comparability across analyses.

## Supplementary material

For supplementary material accompanying this paper visit https://doi.org/10.1017/S0033291719003672. click here to view supplementary material
