# Supplementary material for: Association of etiological factors across the extreme end and continuous variation in disordered eating in female Swedish twins
Source: Psychol Med. 2019 Dec 17;51(5):750–60. doi: 10.1017/S0033291719003672 (PMC8108395; doi:10.1017/S0033291719003672)
Supplement: Supplementary file 1 [file S0033291719003672sup001.docx]

**ONLINE SUPPLEMENTARY MATERIAL**

**Supplementary** **Table S1** Assumption testing of the twin model for the log-transformed Eating Disorder Inventory-2 (EDI-2) score in the full sample

**Supplementary** **Table S2.** Number of twin pairs concordant and discordant for any eating disorder (ED), anorexia nervosa (AN) and other eating disorder (OED) in the joint categorical-continuous models

**Supplementary** **Table S3.** Estimates from the joint categorical-continuous models for the log-transformed Eating Disorder Inventory-2 (EDI-2) score and eating disorder diagnoses

**Supplementary** **Table S1** Assumption testing of the twin model for the log-transformed Eating Disorder Inventory-2 (EDI-2) score in the full sample

| Model | -2LL | Parameters | df | Δχ^2^ | Δdf | p | AIC | BIC |
| --- | --- | --- | --- | --- | --- | --- | --- | --- |
| Fully Saturated | 7826.84 | 10 | 2952 | ----- | ----- | ----- | 1922.84 | -13724.16 |
| Submodel 1 | 7829.84 | 8 | 2954 | 3.01 | 2 | 0.22 | 1921.84 | -13735.75 |
| Submodel 2 | 7829.89 | 6 | 2956 | 3.05 | 4 | 0.55 | 1917.89 | -13750.31 |
| Submodel 3 | 7833.83 | 5 | 2957 | 7.00 | 5 | 0.22 | 1919.83 | -13753.66 |
| Submodel 4 | 7835.36 | 4 | 2958 | 8.53 | 6 | 0.20 | 1919.36 | -13753.66 |

-2LL, -2LogLikelihood; df, degrees of freedom; Δχ2, difference in -2LL between two models, distributed χ2; Δdf, difference in degrees of freedom between two models; p, p-value from likelihood-ratio tests, AIC, Akaike Information Criterion; BIC, Bayesian Information Criterion.
*Note.* All submodels are compared with the fully saturated model. Submodel 1 tests equal means across twin order within zygosity group. Submodel 2 tests equal means and variances across twin order within zygosity group. Submodel 3 tests equal means and variances across twin order within zygosity group, and equal means across zygosity group. Submodel 4 tests equal means and variances across twin order and across zygosity group.

|  |  |  |  |
| --- | --- | --- | --- |

**Supplementary** **Table S2** Number of twin pairs concordant and discordant for any eating disorder (ED), anorexia nervosa (AN) and other eating disorder (OED) in the joint categorical-continuous models

|  | MZ  n=768 pairs | | DZ  n=713 pairs | |
| --- | --- | --- | --- | --- |
|  | Concordant | Discordant | Concordant | Discordant |
|  | n (%) | n (%) | n (%) | n (%) |
| ED | 20 (2.6) | 58 (7.5) | 1 (0.1) | 69 (9.7) |
| AN | 7 (0.9) | 25 (3.3) | 0 (0.0) | 31 (4.3) |
| OED | 11 (1.4) | 37 (4.8) | 1 (0.1) | 38 (5.3) |

MZ, monozygotic twins; DZ, dizygotic twins

| **Supplementary** **Table S3** Estimates from the joint categorical-continuous models for the log-transformed Eating Disorder Inventory-2 (EDI-2) score and eating disorder diagnoses | | | | | | | | | | | | | |  |
| --- | --- | --- | --- | --- | --- | --- | --- | --- | --- | --- | --- | --- | --- | --- |
|  |  |  |  |  |  |  |  |  |  |  |  |  |  | |
|  | EDI-2 | | | ED/AN/OED | | | Correlations | | | | Proportion of r_Ph_ explained by genes and environment | | | |
|  | A | C | E | A | C | E | rPH | rA | rC | rE | A | C | E | |
| ED | 0.65 (0.61-0.68) | ----- | 0.35 (0.32-0.39) | 0.62 (0.60-0.64) | ----- | 0.38 (0.36-0.40) | 0.52 (0.46-0.58) | 0.48 (0.36-0.59) | ----- | 0.60 (0.45-0.73) | 0.58 (0.54-0.62) | ----- | 0.42 (0.38-0.46) | |
| AN | 0.65 (0.61-0.68) | ----- | 0.35 (0.32-0.39) | 0.63 (0.39-0.81) | ----- | 0.37 (0.19-0.61) | 0.39 (0.29-0.48) | 0.26 (0.08-0.42) | ----- | 0.60 (0.38-0.79) | 0.43 (0.14-0.67) | ----- | 0.57 (0.33-0.86) | |
| OED | 0.65 (0.61-0.68) | ----- | 0.35 (0.32-0.39) | 0.67 (0.49-0.81) | ----- | 0.33 (0.19-0.51) | 0.52 (0.44-0.60) | 0.52 (0.39-0.65) | ----- | 0.52 (0.32-0.70) | 0.66 (0.51-0.80) | ----- | 0.34 (0.20-0.49) | |
| ED, any eating disorder; AN, anorexia nervosa; OED, other eating disorder; EDI-2, Eating Disorder Inventory-2; A, additive genetic influences; C, shared environmental influences; E, non-shared environmental influences, rA, additive genetic correlation; rC, shared environmental correlation; rE, non-shared environmental correlation; rPh, phenotypic correlation. | | | | | | | | | | | | | | |
